# Automatic deep learning method for third lumbar selection and body composition evaluation on CT scans of cancer patients

**DOI:** 10.3389/fnume.2023.1292676

**Published:** 2024-01-10

**Authors:** Lidia Delrieu, Damien Blanc, Amine Bouhamama, Fabien Reyal, Frank Pilleul, Victor Racine, Anne Sophie Hamy, Hugo Crochet, Timothée Marchal, Pierre Etienne Heudel

**Affiliations:** ^1^ Residual Tumor & Response to Treatment Laboratory, RT2Lab, Translational Research Department, INSERM, U932 Immunity and Cancer, Institut Curie, Paris University, Paris, France; ^2^ QuantaCell, Pessac, France; ^3^ IMAG, Université de Montpellier, Montpellier, France; ^4^ Department of Radiology, Centre Léon Bérard, Lyon, France; ^5^ Department of Surgical Oncology, Institut Curie, University Paris, Paris, France; ^6^ Department of Medical Oncology, Institut Curie, University Paris, Paris, France; ^7^ Data and Artificial Intelligence Team, Centre Léon Bérard, Lyon, France; ^8^ Department of Supportive Care, Institut Curie, Paris, France; ^9^ Department of Medical Oncology, Centre Léon Bérard, Lyon, France

**Keywords:** body composition, computed tomography, deep learning, sarcopenia, cancer

## Abstract

**Introduction:**

The importance of body composition and sarcopenia is well-recognized in cancer patient outcomes and treatment tolerance, yet routine evaluations are rare due to their time-intensive nature. While CT scans provide accurate measurements, they depend on manual processes. We developed and validated a deep learning algorithm to automatically select and segment abdominal muscles [SM], visceral fat [VAT], and subcutaneous fat [SAT] on CT scans.

**Materials and Methods:**

A total of 352 CT scans were collected from two cancer centers. The detection of the third lumbar vertebra and three different body tissues (SM, VAT, and SAT) were annotated manually. The 5-fold cross-validation method was used to develop the algorithm and validate its performance on the training cohort. The results were validated on an external, independent group of CT scans.

**Results:**

The algorithm for automatic L3 slice selection had a mean absolute error of 4 mm for the internal validation dataset and 5.5 mm for the external validation dataset. The median DICE similarity coefficient for body composition was 0.94 for SM, 0.93 for VAT, and 0.86 for SAT in the internal validation dataset, whereas it was 0.93 for SM, 0.93 for VAT, and 0.85 for SAT in the external validation dataset. There were high correlation scores with sarcopenia metrics in both internal and external validation datasets.

**Conclusions:**

Our deep learning algorithm facilitates routine research use and could be integrated into electronic patient records, enhancing care through better monitoring and the incorporation of targeted supportive measures like exercise and nutrition.

## 1 Introduction

Body composition plays a crucial role in the development and progression of numerous diseases, including cancer (1–3). The prevalence of the decrease in muscle mass, known as sarcopenia, varies depending on the cancer stages, ranging from 39.6% for curable cancers to 49.2% in palliative conditions (4). In 2023, a study that included 20 meta-analyses involving over 52,600 patients revealed that sarcopenia was predictive of both overall and disease-specific survival in various cancer types, such as lung and digestive cancer (5). Its assessment has been of growing interest in recent years, as low muscle mass is also predictive of chemotherapy toxicity (6–15), dose-limiting toxicities (16), and poor prognosis (17–19).

Despite its significant impact on cancer patient care, body composition evaluation is not performed routinely or used in clinical decision-making (20). Imaging techniques such as dual-energy x-ray absorptiometry and computed tomography (CT) scans are highly accurate methods for assessing body composition. In the oncology setting, CT scan cross-section at the third lumbar (L3) level is widely used for this purpose as part of routine cancer diagnostic procedures with no additional cost or toxicities (16, 21–24, 25). However, manual measurement of body composition through CT scans is time-consuming and requires expertise, which limits its practical use in daily clinical practice. Artificial intelligence using deep learning provides an opportunity to automate muscle mass assessment with high precision (26–31), but validated tools for research and integration into the electronic medical record are scarce. Accordingly, studies proposing algorithms for body composition detection from L3 have been conducted in homogeneous populations with few challenging cases, such as patients undergoing cementoplasty or those with intra-corporeal devices, which are common in the cancer population, especially in metastatic patients. A study conducted by Ha focused on the development of a deep learning model for L3 slice selection and a fully convolutional network (FCN)-based algorithm for abdominal muscle and fat segmentation (30). The study demonstrated high accuracy in automatic L3 slice selection, with mean distance differences of 3.7 ± 8.4 mm and 4.1 ± 8.3 mm in internal and external validation datasets, respectively. However, challenges arose in cases involving anatomic variations, highlighting the need for improved methods in these scenarios. Kreher presented a deep learning-based approach for skeletal muscle mass segmentation at the L3 level in routine abdominal CT scans (32). Utilizing a U-Net architecture, the study achieved impressive Dice scores ranging from 0.86 to 0.95 for different muscle types. This approach demonstrated the potential to expedite the segmentation process and serve as a foundation for future biomarker development. A recent meta-analysis published in 2023 assessed the feasibility and accuracy of automatic segmentation tools for body composition through 92 studies (33). The review highlighted the success of deep learning algorithms in achieving excellent segmentation performance, especially in the context of rapid and automated volumetric body composition analysis. However, the study emphasized the need for consensus in defining accuracy and precision standards for ground-truth labeling, ensuring the reliability of these automated techniques. While these studies have undoubtedly advanced the field of automated abdominal muscle assessment and body composition analysis, they have also highlighted the need for continuous research particularly in the face of challenges related to anatomic variations, segmentation accuracy, and standardization of protocols. More studies are needed to contribute significantly to the field by collecting international data on segmentation algorithms. This collaborative effort will undoubtedly propel the field of abdominal muscle assessment through CT imaging to unprecedented heights.

The main aim of this study was to develop and validate a deep learning-based algorithm to automatically select the L3 slice on abdominal CT scans of cancer patients and segment abdominal muscles (SM), visceral fat (VAT), and subcutaneous fat (SAT).

## 2 Materials and methods

### 2.1 Ethics

The study was approved in July 2020 by the local data protection officer on behalf of French regulatory authorities (Commission Nationale de l'Informatique et des Libertés, CNIL) in accordance with MR004 methodology (R201-004-207). All patients were informed of the possibility of their health data being used for research purposes, and they expressed no objections to this possibility.

A MedExprim® tool (34) was used to obtain the images of the patients, which were exported in batches from PACS and de-identified.

### 2.2 Study design

**Figure 1:**
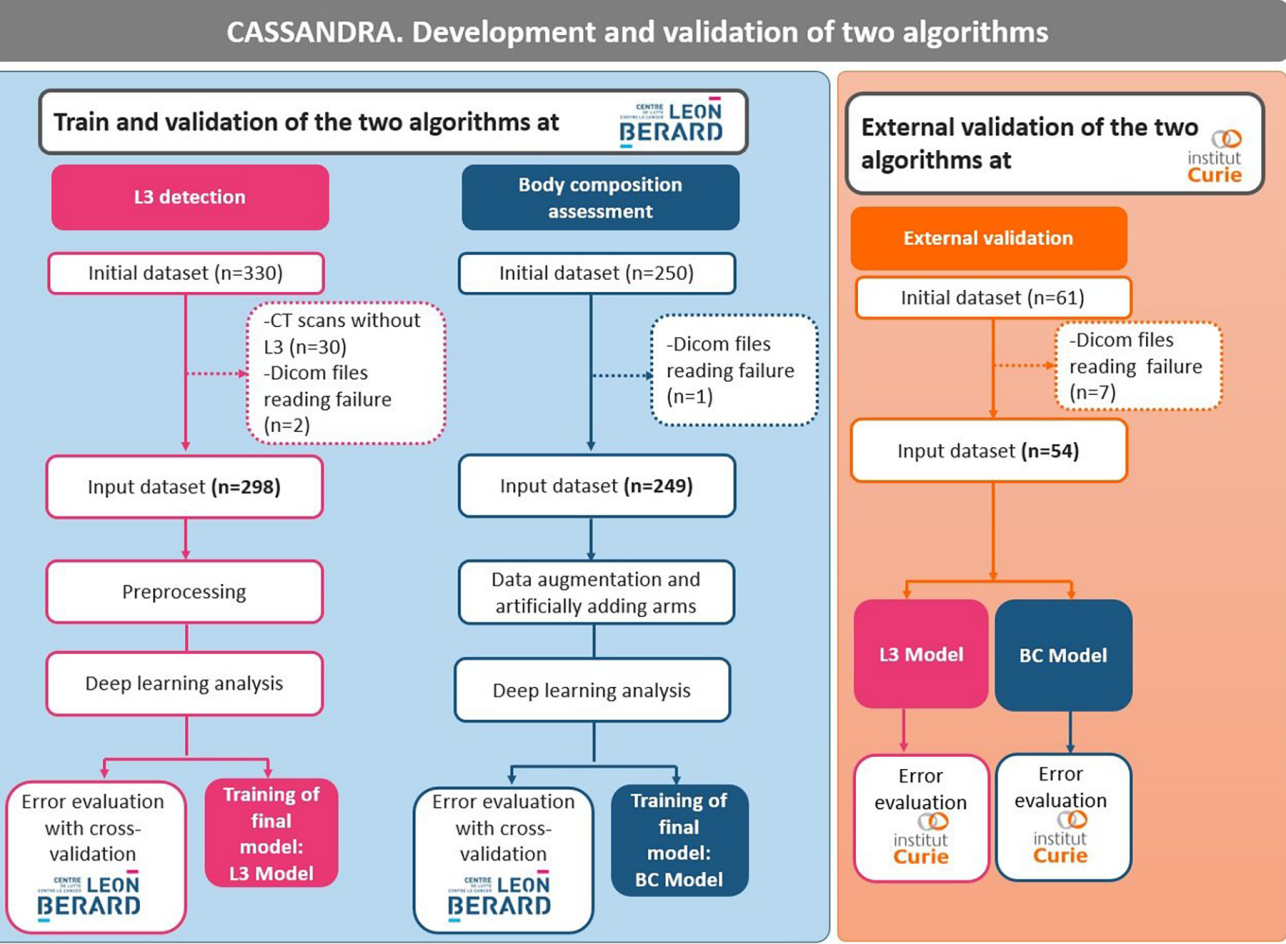
Composition of the datasets.

Three datasets were used to: (i) develop an algorithm to select L3 (dataset 1); (ii) develop an algorithm to segment body composition (dataset 2); and (iii) validate both algorithms on an external validation cohort (dataset 3) (Figure 1). The development of the two algorithms was carried out on patients with solid cancers treated with immunotherapy at the Leon Bérard Center in Lyon. External validation in patients with solid tumors from the Institut Curie, Paris, was also carried out.

As the two cancer facilities are both regional comprehensive cancer centers, a sense of data diversity (e.g., scanners, slice thickness, resolution) was obtained, which reinforces the robustness and generalization of the algorithms. All images were included, even those with artifacts, arms in the field of view, spinal cementoplasty, or other intracorporeal devices (ureteral stents, for example).

### 2.3 Patient characteristics

**Dataset 1** for L3 selection consisted of a total of 330 cancer patients (Table 1). Out of this population, 30 subjects were excluded because the scans did not contain the L3 slice and two because of problems reading the DICOM file. The final dataset was composed of 298 cancer patients [137 women and 161 men, mean age 59.9 years (range: 18‒96)]. All patients had CT scans. A total of 286 patients had metastatic cancer, and 25 had multiple cancers.

**Table 1:** Clinical characteristics of the study population.

	Centre Léon Bérard		Institut Curie
	Dataset 1	Dataset 2	Dataset 3
	L3 selection (*n* = 298)	Body composition segmentation (*n* = 249)	External validation (*n* = 54)
Age (years), mean (±SD)	59.9 ± 11.8	58.0 ± 12.2	61.8 ± 10.9
Sex, *n* (%)			
Female	137 (45.9)	89 (35.7)	23 (42.6)
Male	161 (54.1)	160 (64.2)	31 (57.4)
BMI (kg/m^2^), *n* (%)^a^			
Underweight	29 (11.7)	26 (10.1)	2 (3.7)
Normal	131 (52.8)	148 (57.4)	32 (59.3)
Overweight	66 (26.6)	62 (24.0)	18 (33.3)
Obese	22 (8.9)	22 (8.5)	2 (3.7)
Metastasis, *n* (%)	286 (95.9)	224 (89.9)	NA
More than one cancer, *n* (%)	25 (8.3)	15 (6)	NA
Solid tumor, *n* (%)	298 (100)	248 (100)	54 (100)

^a^ Missing data for L3 selection (*n* = 35).

**Dataset 2** for body composition included 250 patients and one patient had a DICOM file reading error. Thus, the final database consisted of 249 cancer patients [89 women and 160 men; mean age 58.0 years (range: 18‒85)] (Table 1). All patients had solid cancers, 224 had metastatic cancers, and 15 had multiple cancers.

**Dataset 3** for the external validation of the algorithm originally consisted of 60 cancer patients (Table 1). However, the DICOM files of seven patients could not be opened, resulting in a final database of 53 patients (all women, mean age 61.8 years) treated for either metastatic breast cancer or lung cancer.

### 2.4 Manual labeling and data format

All the images were labeled by a senior radiologist (AB) to validate the L3 location and the body composition segmentation.

#### 2.4.1 L3 selection

The DICOM-format axial slices of a CT scan were evaluated to identify L3, which was then located and recorded in an Excel table (.xlsx), indicating its position in the full 3D scan.

#### 2.4.2 Body composition segmentation

To segment the L3 scan sections manually, 3D slicer was used. The segmentation process involved two steps: (i) an initial segmentation was performed using intensity thresholds; (ii) a manual pixel-by-pixel correction was then performed for each class. To segment the muscle tissue, an intensity threshold ranging from −29 to 150 Hounsfield units (HU) was applied. Similarly, for adipose tissue, which includes both SAT and VAT, an intensity threshold ranging from −500 to −30 HU was used (35).

#### 2.4.3 Data preprocessing

To simplify the task and reduce resource requirements, several preprocessing steps were undertaken. A maximum Z-projection was applied to all 3D scans, resulting in a 2D coronal slice where L3 was typically visible. Intensity thresholding was then performed, followed by morphological mathematical operations to obtain a whole-body segmentation mask.

Mathematical operations, such as dilation and closing, are fundamental image analysis techniques widely used to smooth and shape regions in binary or grayscale images. The voxels outside of this mask were reduced to zero before projection. Next, a pre-cropping procedure was performed on each scan to eliminate artifacts in irrelevant regions and to reduce the image dimensions as much as possible. The procedure consisted of two steps: (i) rough localization of the lung centroid based on an intensity threshold; followed by (ii) localization of the pelvic centroid using a skeletonization method on a bone mask. However, this method was not applied in all situations, and pre-cropping was only done when the resulting distance between the two centroids exceeded 20 cm. To standardize the dimensions, the entire dataset was scaled to 1 mm thickness per pixel, and all images were padded to achieve a size of 1064 × 512. The L3 detection problem was treated as a segmentation task, and thus a binary mask with a 1-pixel-thick region was used to locate L3 after the preprocessing step. To partially address the class imbalance, the L3 region was extended to a thickness of 1 cm.

#### 2.4.4 Training procedure

A popular deep learning model for medical image segmentation is U-net, which is designed for semantic segmentation tasks and has been successfully applied to body composition analysis (26, 27, 32, 36). The U-net architecture includes a contracting path for feature extraction and a symmetric expanding path for precise localization, which allows for accurate segmentation of complex anatomic structures. The U-net model specifications were carefully designed to optimize the performance of our system. We utilized a standard U-net architecture with 3 × 3 convolutional kernels and structured the model with an encoder and decoder block of layers. Our architecture comprised four down-sampling and up-sampling layers, ensuring a robust and effective feature extraction process. To fine-tune our approach, we meticulously adjusted the number of filters in each convolutional layer using a progressive increase/decrease strategy. For the L3 detection task, we initiated the first layer with 64 filters and doubled the number of filters in subsequent layers, whereas for the body composition segmentation, a relatively less complex task, we started with 32 filters. Notably, our models were initialized with random weights, although we acknowledge the potential advantages of utilizing pre-trained models, especially on larger datasets. To measure the dissimilarity between predicted and ground truth segmentations, we employed the dice loss function, a common choice for segmentation tasks. Additionally, we paid close attention to the input size, padding the images to 1088 × 512 pixels for L3 detection and setting it to 512 × 512 pixels for body segmentation. This preprocessing step was crucial in adapting the images to the specific requirements of each task, ensuring accurate and reliable results. Our study showcases the careful consideration of model specifications, pre-training strategies, loss functions, and input size adjustments, highlighting the meticulous approach taken to achieve superior performance in medical image analysis using deep learning techniques.

To increase the size and variability of our training dataset and prevent overfitting, geometric data augmentation techniques were used. These techniques involved applying various transformations to the CT scans, such as rotation, scaling, translation, and elastic deformations, to generate new images with different orientations, scales, positions, and more realistic deformations. We also added artificial sections of the arms to the L3 slices using intensity-based thresholding, varying their positions and orientations to enhance the variability of the training dataset. Supplementary Figure S1 shows examples of different data augmentation techniques applied to a single slice. This resulted in a much larger and more diverse training dataset, allowing deep learning models to learn more robust features from the data. The effectiveness of this technique was evaluated by comparing the performance of the U-net model with and without the additional arm sections, and the results showed that the inclusion of the augmented images significantly improved the accuracy and robustness of the U-net model. These findings suggest that this data augmentation technique could be useful for improving the performance of convolutional neural networks (CNNs) in body composition analysis.

Two U-net models were trained for both L3 detection and body composition separately. The training was performed using the Adam optimizer with a learning rate of 0.0001 and a batch size of 8. Models were trained for 100 epochs on the augmented dataset, with early stopping based on the validation loss. During training, the model was evaluated on the validation set after each epoch using the DICE coefficient, and the best-performing model was saved for testing. The final trained model was evaluated on the test set to measure its generalization performance.

#### 2.4.5 Testing procedure and post-processing

Both L3 detection and body composition problems were treated as segmentation problems. Therefore, each prediction was a probability map, where each pixel was assigned a probability of belonging to a certain class. In the case of L3 detection, the two classes were the L3 slice and the rest of the body, while for body composition segmentation, the classes corresponded to different tissue types (e.g., adipose tissue, muscle tissue, etc.).

For the L3 detection task, the final slice value was obtained through a post-processing step involving a projection of the probability map. Specifically, the probability map was projected onto a one-dimensional signal by taking the average probability along the vertical axis. This signal was then smoothed using a Gaussian filter to reduce the noise. The predicted L3 slice location was then determined by identifying local maxima in the smoothed signal, where the location of the global maximum corresponded to the precise location of the L3 slice.

To further refine the body composition segmentation results, a post-processing step was applied to the predicted probability maps for each class. This step involved thresholding the probability maps based on the HU values of the corresponding pixels. All pixels with HU values >150 or <−500 were ignored, as they were considered to be outside the range of interest. This helped to remove any noise or outliers in the predictions and improve the accuracy of the segmentation results.

#### 2.4.6 Sarcopenia measurements

Skeletal muscle density (SMD) was quantified as the mean muscle radiation attenuation (in HU) of the muscle cross-sectional area across the L3 vertebral body level and was assessed between −29 and 150 HU (37). Skeletal muscle index (SMI) (cm^2^/m^2^) was obtained by normalizing muscle cross-sectional area to patient height. Skeletal muscle gauge (SMG) (HU × cm^2^/m^2^) was calculated by multiplying muscle area by SMD. Lean body mass (LBM) was calculated according to the following equation: LBM (kg) = 0.3 × muscle cross-sectional area at L3 (cm^2^) + 6.06 (25).

#### 2.4.7 Performance evaluation

To evaluate the performance of the U-net model for L3 detection and body composition segmentation, a cross-validation approach was used. Cross-validation is a commonly used technique to assess the generalizability of a model by dividing the dataset into training and testing sets and repeating the process multiple times with different partitions of the data. In this study, a 5-fold cross-validation was employed on datasets 1 and 2 to evaluate the robustness and accuracy of the U-net model. After the 5-fold cross-validation, the models were fully trained on datasets 1 and 2 and tested on dataset 3, which was not used for training.

The evaluation of the proposed algorithms was performed on several levels, including accurate detection of L3, precise segmentation of body composition, and reliable measurement of sarcopenia. Therefore, the performance of the models was evaluated using several metrics, including the mean absolute error (MAE), the DICE coefficient, the mean absolute percentage error (MAPE), and the *R*^2^ coefficient.

MAE is a suitable metric to measure the distance between the predicted location and the ground truth location of L3. By using MAE, the accuracy of the model at predicting the exact location of L3 can be evaluated, regardless of whether the prediction is slightly above or below the true value. The closer the MAE is to 0, the more accurate the model is.

The DICE coefficient was used to evaluate the agreement between measurements from fully automatic measurement and manual segmentation. This metric measures the overlap between the predicted and ground-truth segmentation masks (0 = no overlap; 1 = perfect overlap). Our results demonstrate that the U-net model achieved high accuracy and robustness across different datasets, indicating its potential as a reliable tool for body composition analysis in clinical practice.

MAPE measures the percentage difference between the predicted and ground-truth values, which is particularly useful when dealing with relative measurements such as muscle mass or muscle area.

The *R*^2^ score is a measure of how well the predicted values match the ground-truth values and ranges from 0 to 1, with higher values indicating better performance. These metrics provide additional insights into the accuracy and reliability of the sarcopenia measurements produced by the deep learning models, beyond the traditional MAE metric used for L3 detection.

To compare the performance of manual vs. automatic segmentation, three methods were applied: MAE, MAPE, and *R*^2^ coefficient. An *R*^2^ coefficient of >0.7 would generally be seen as showing a high level of correlation, whereas a value <0.4 would show a low correlation.

All analysis was performed using Python (version 3.9) and the Tensorflow (version 2.5.0) library with a GeForce 2080Ti GPU.

## 3 Results

### 3.1 Evaluation of L3 slice selection

**Figure 2:**
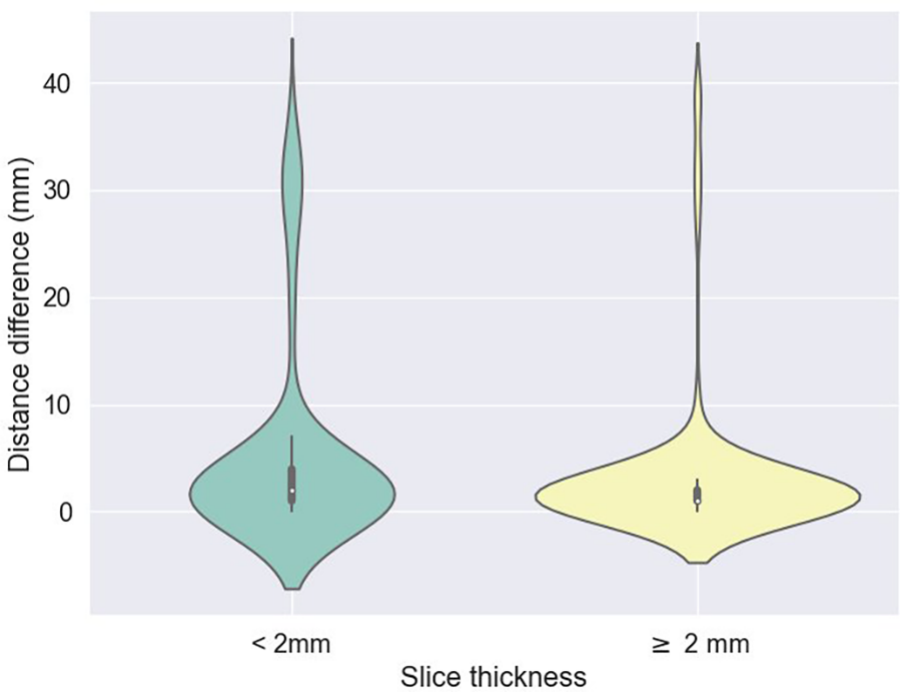
Difference between predicted (z) values by the L3 detection model and ground truth, stratified by scan thickness. The mean differences between the ground truth and the model predictions were 5.2 mm ± 9.1 for scans less than 2 mm thick and 3.1 mm ± 6.7 for scans 2 mm or thicker.

The accuracy of the algorithm for automatic L3 slice selection in the internal and external validation (datasets 1 and 3, respectively) is presented in Figure 2.

The mean absolute errors in slice selection between manual and automated L3 slice selection were 4.0 mm (±9.6) and 5.5 mm (±7.8) for the internal and external validation, respectively (datasets 1 and 3, respectively). The height of the vertebral body is approximately 40 mm (30).

**Figure 3:**
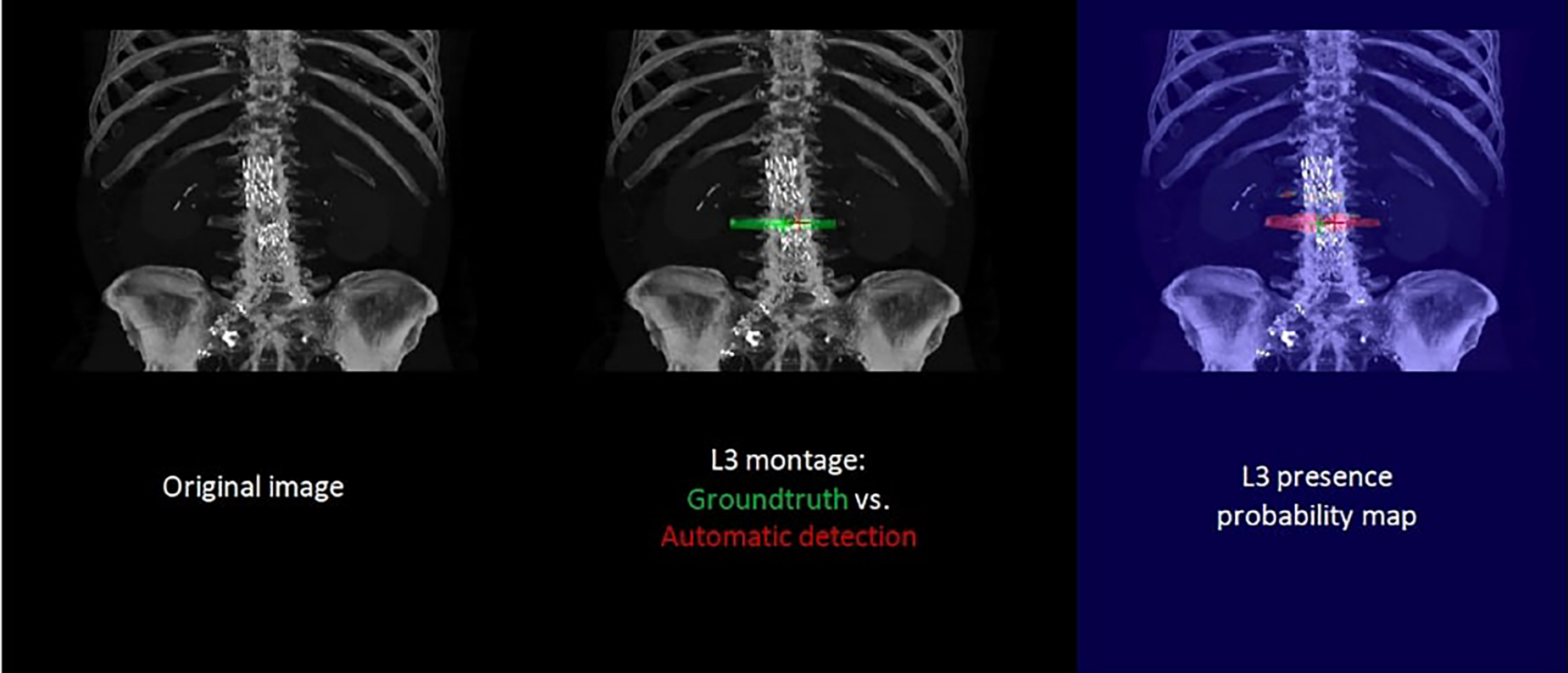
Example of manual (green) and automatic (red) detection.

In the majority of cases, automated segmentation correctly identified the L3 slice for 91.2% and 74.1% in datasets 1 and 3, respectively (Figure 3). Patients with intracorporeal devices or spinal changes had scores of 7.0 mm (±10.9) for dataset 1 (*n* = 31) and 5.0 mm (±6.2) for dataset 3 (*n* = 11).

### 3.2 Evaluation of body composition

**Figure 4:**
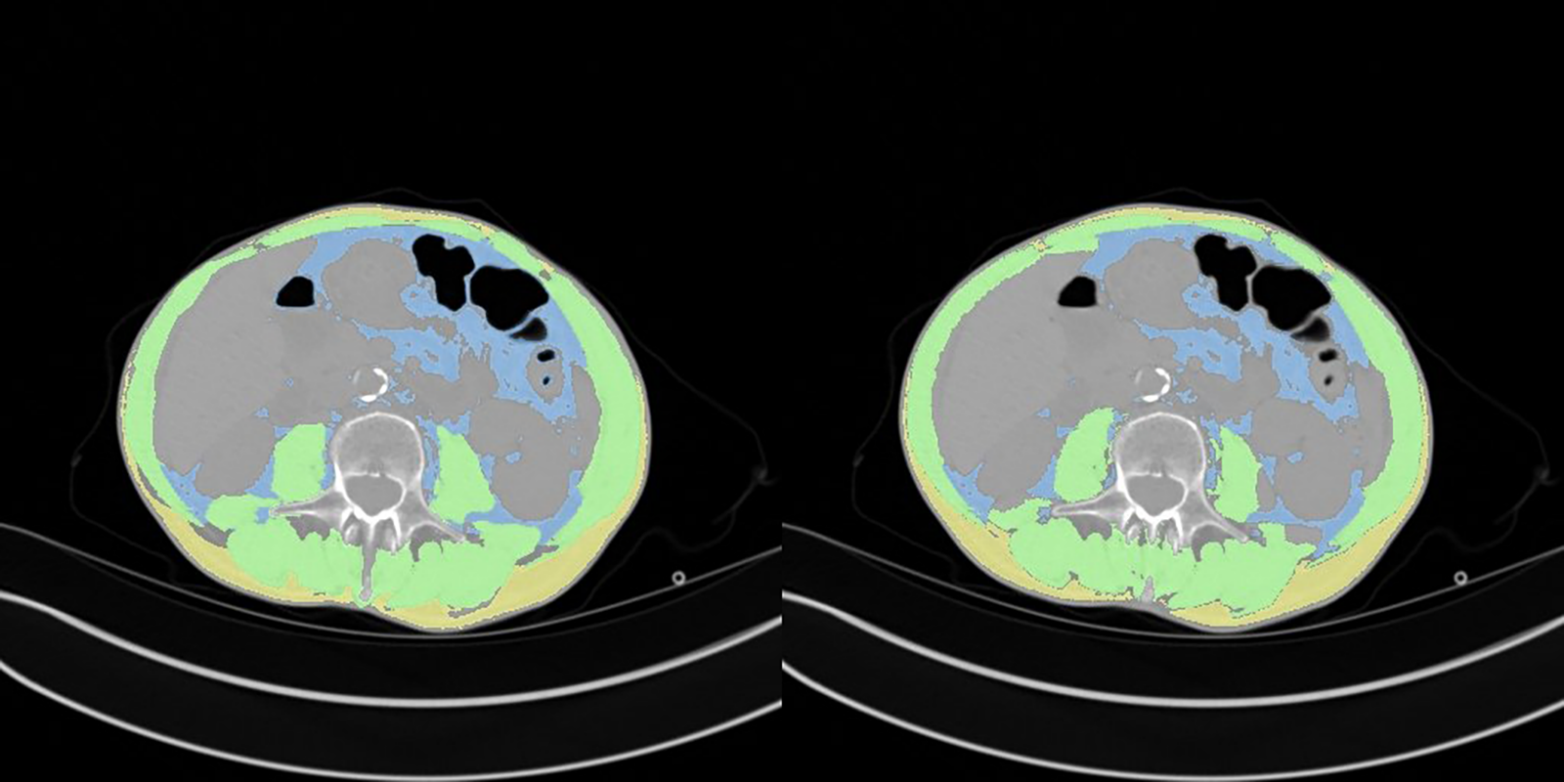
Comparison between manual (left) and automatic segmentation (right).

A comparison between manual and automatic segmentation is shown in Figure 4. The median DICE similarity coefficient (DSC) (and interquartile range) indicated excellent overlap >0.85 for both internal and external validation datasets (datasets 2 and 3, respectively) (Table 2). In dataset 2, our model had a DSC of 0.937 for SM and 0.927 for VAT. Similar results were found for dataset 3 with a DSC of 0.933 for SM and 0.930 for VAT. The DSC was lowest for the measurement of SAT in both datasets 2 and 3 (0.855 and 0.850, respectively).

**Table 2:** Performance of the model on the validation sets.

	Dataset 2	Dataset 3
	DICE similarity coefficient	DICE similarity coefficient
Background	1.0 ± 0.00	1.0 ± 0.00
Body	0.91 ± 0.06	0.91 ± 0.06
Muscle (SM)	0.94 ± 0.06	0.93 ± 0.13
Visceral fat (VAT)	0.93 ± 0.14	0.93 ± 0.16
Subcutaneous fat (SAT)	0.86 ± 0.16	0.85 ± 0.18

### 3.3 Correlation between body composition and sarcopenia measurements

The correlations between the predicted and manually performed areas for dataset 2 were 0.79 for SM, 0.98 for VAT, and 0.98 for SAT (Table 3). For dataset 3, the results were 0.95 for SM, 1.0 for VAT, and 0.99 for SAT. A high correlation score with sarcopenia metrics was observed (SMD: 0.94, SMI: 0.73, LBM: 0.75, SMG: 0.93). High correlation scores with sarcopenia metrics were also found for both dataset 2 and dataset 3.

**Table 3:** Correlation of Body Mass Index and Body Composition assessments in internal and external validation datasets.

	Internal validation dataset	External validation dataset
Muscle surface (SM) estimation (mm^2^)		
*R* ^2^	0.79	0.95
MAE	886.6	530.7
MAPE	9.2	4.3
Visceral fat surface (VAT) estimation (mm^2^)		
*R* ^2^	0.98	1.0
MAE	960.7	588.6
MAPE	10.6	4.1
Subcutaneous fat surface (SAT) estimation (mm^2^)		
*R* ^2^	0.98	0.99
MAE	987.3	645.4
MAPE	15.7	6.9
Skeletal muscle density (SMD)		
*R* ^2^	0.94	0.91
MAE	1.3	2.5
MAPE	4.1	8.4
Skeletal muscle index (SMI)		
*R* ^2^	0.73	0.95
MAE	3.0	1.8
MAPE	9.1	4.3
Lean body mass (LBM)		
*R* ^2^	0.79	0.95
MAE	266.0	159.2
MAPE	9.2	4.3
Skeletal muscle gauge (SMG)		
*R* ^2^	0.93	0.94
MAE	97.9	81.6
MAPE	9.0	6.5

## 4 Discussion

This study has addressed a critical issue in the field of automated body composition analysis by focusing on the automatic segmentation of L3 slices and developing a robust model for the detection of skeletal muscle, visceral adipose tissue, and subcutaneous adipose tissue. We obtained highly accurate results for CT scans of L3 slices and automatic segmentation, as demonstrated by our internal and external validation datasets.

Our findings regarding the automatic selection of L3 are consistent with previous studies in the literature (3, 30, 38). While some studies might demonstrate slightly higher performance metrics, the reliability and stability of our results across diverse datasets underscore the robustness of our algorithm. In a study of 922 individuals, the mean distance difference between ground-truth and deep learning model-derived L3 slices was 3.7 and 4.1 mm for the internal (30) and external validation cohorts, respectively whereas in our study it was 4 and 5.5 mm, respectively.

Despite the importance of sarcopenia as a potential target in oncology, routine data in this area is lacking (5). Analyzing body composition is a challenging task due to the varied methods used (39). When comparing similar populations who have undergone automated body composition analysis at the L3 level, the average DICE scores for SAT, VAT, and SM are all >0.90 (26, 30, 32, 33, 40–45), which is very similar to our study. Some studies have attempted to identify lipid infiltration within muscle as an indicator of muscle quality, but the difficulty is obtaining accurate measurements, and its clinical relevance remains uncertain (27, 46).

Body composition assessment through CT scans, especially at the L3 level, is increasingly utilized in the field of research. The advantage lies in the ability to conduct analyses at the time of diagnosis, and during follow-ups, both longitudinally and retrospectively. In the majority of studies, body composition assessment is performed at the level of L3 (47–50) and sometimes L4 (51, 52). Another study showed that abdominal muscle surface area did not differ significantly between L3 and the lower part of L2 or L4, suggesting the possibility of considering margins slightly larger than 40 mm (53). Some authors have highlighted the need to find other methods that are more representative than the L3 slice to estimate participants’ body composition, as indicated by Pu et al. in 2023 (26, 27, 30, 40–45). A meta-analysis published in 2022 indicated that skeletal muscle index thresholds at the L3 level ranged from 52 to 55 cm/m^2^ for men and from 39 to 41 for women (54). However, due to the lack of consensus in the literature, multiple sarcopenia thresholds exist based on different vertebral locations, as studied by Derstine et al. (55). Nevertheless, a systematic review of 388 articles revealed that the L3 level is most commonly used to measure body composition (54). By adhering to the widely accepted L3 standard, our study contributes to the establishment of a unified reference framework, enabling meaningful comparisons and comprehensiveness.

To overcome the challenges of heterogeneity in the literature, our study offers a pivotal contribution to the integration of artificial intelligence in the domain of body composition assessment. The development of validated automatic segmentation algorithms facilitates a novel frontier for both clinical application and investigative inquiry. Such advancements herald a transformative potential for individualized patient management through enhanced early detection of body composition variations. Our work not only enriches the data landscape, fostering a deeper comprehension of cancer demographics, but also lays the groundwork for establishing robust benchmarks, constructing predictive models for health outcomes, and potentially refining treatment dosing protocols. In particular, incorporating body composition metrics into clinical trial inclusion criteria may revolutionize the administration of oncological therapies (56). Tailoring drug regimens to each patient's unique physiological makeup could significantly refine therapeutic efficacy and safety, marking a new era in personalized medicine. Our research endeavors are therefore crucial stepping stones toward a more nuanced and effective healthcare paradigm.

Our study has several limitations. The algorithm was developed using CT scan images of patients with solid tumors only. Increasing the sample size to include more patients with intracorporeal devices and cementoplasty in particular would also improve the model's performance and increase the heterogeneity of the data. It would be intriguing to further train the model with scenarios that are particularly unique to oncology, such as patients with ascites, extensive peritoneal carcinomatosis, or significant retroperitoneal lymph node involvement as seen in hematological malignancies. Furthermore, our algorithm does not enable the analysis of body composition for patients with chest imaging, such as head and neck cancers. Several ongoing projects utilizing our algorithm aim to: (i) investigate the associations between body composition and the survival of patients treated with immunotherapy, following an article published with body mass index; (ii) study the longitudinal changes in body composition from multiple time points; and (iii) develop predictive models for toxicity under immunotherapy.

Our manuscript marks a significant advancement in automated body composition analysis, a field currently facing a lack of robust, validated tools for routine clinical application. By meticulously validating our automated method against established manual techniques, we establish its precision and reliability, ensuring that it stands up to the rigorous demands of clinical practice. The diversity of the image data, sourced from various machines and institutions, speaks to the robustness of our algorithm. It skillfully navigates the challenges of image analysis, discerning relevant anatomical features with the ability to isolate the L3 slice while excluding non-pertinent elements, such as arms that may confound the assessment. This level of precision in automated body composition analysis is not just a theoretical enhancement; it is a practical tool poised for integration into clinical workflows. It promises to enrich research, refine patient monitoring, and facilitate the direct incorporation of holistic supportive care measures—including targeted nutrition and exercise regimens— into patient management plans. The possibility of integrating this algorithm into electronic health records represents a transformative step forward in the personalization and optimization of cancer care.

In conclusion, our study presents a sophisticated algorithm capable of autonomously detecting the L3 vertebra and delineating skeletal muscle, visceral, and subcutaneous adipose tissue compartments. The seamless integration of this tool into both research frameworks and routine clinical practice promises to revolutionize our understanding and utilization of body composition data. By harnessing the capabilities of artificial intelligence, we facilitate a leap forward not just in knowledge acquisition but in the actualization of enhanced, individualized patient management strategies. Our commitment to making this algorithm openly accessible to the scientific community reflects our intent to foster collective progress in this domain. This collaborative approach aims to expedite the translation of research into meaningful improvements in patient care, reinforcing our overarching goal of delivering health innovations that truly matter.

## Data Availability

The raw data supporting the conclusions of this article will be made available by the authors, without undue reservation. Subject to project approval by the project team and a partnership agreement.

## Supplementary material

SUPPLEMENTARY FIGURE S1

Effect of data augmentation on L3 slice: (a) original image; (b) original image with artificial arms added; (c) rotated original image with artificial arms.
