# Supplementary figures and images for: Automatic deep learning method for third lumbar selection and body composition evaluation on CT scans of cancer patients
Source: Front Nucl Med. 2024 Jan 10;3:1292676. doi: 10.3389/fnume.2023.1292676 (PMC11440831; doi:10.3389/fnume.2023.1292676)

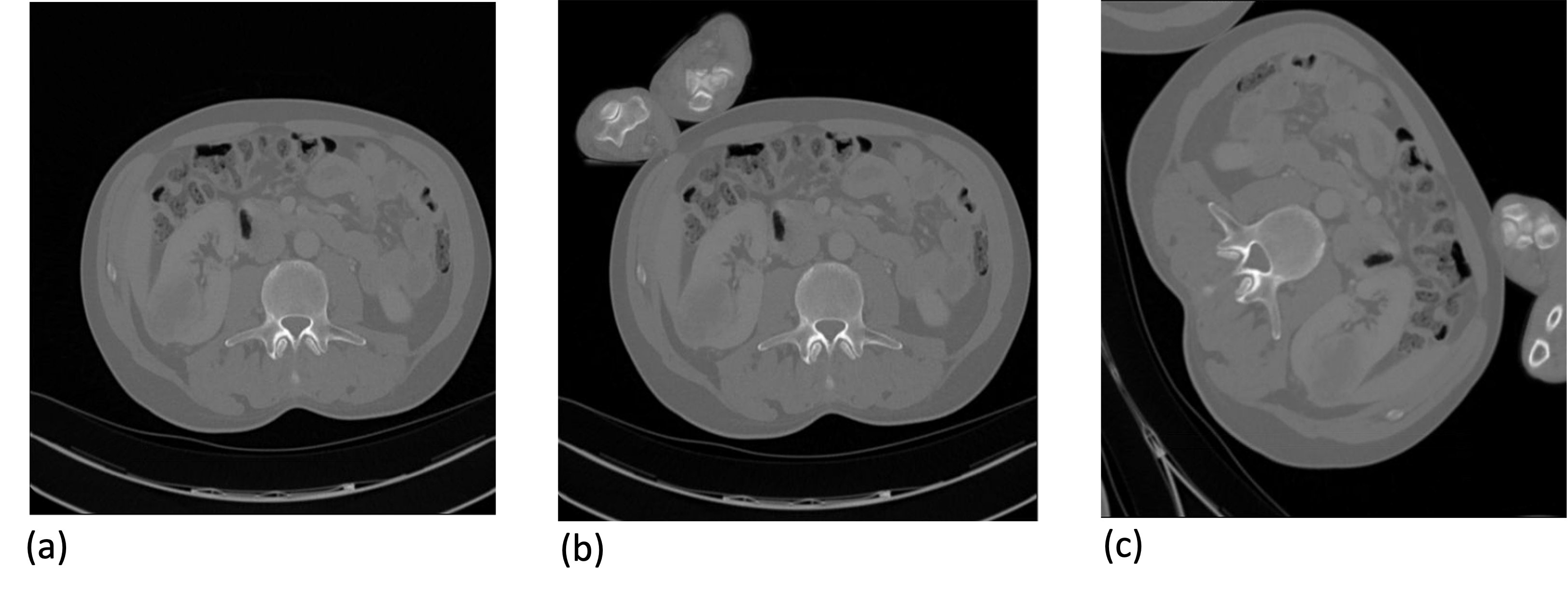

Supplement: Supplementary file 1 [file Image1.jpg]
